# Beyond solute removal: is extracorporeal circuit exposure itself therapeutic in apheresis?

**DOI:** 10.3389/fmed.2026.1944215

**Published:** 2026-10-16

**Authors:** Caitlin Raymond

**Affiliations:** Department of Pathology and Laboratory Medicine, University of Wisconsin–Madison, Madison, WI, United States

**Keywords:** apheresis—donation, apheresis—therapeutic, biocompatibility, extracorporeal circuit, immunomodulation

## Abstract

Therapeutic apheresis is conventionally understood through what it removes: pathogenic antibodies, immune complexes, cytokines, or lipoproteins. This framework treats the extracorporeal circuit as a biologically inert delivery mechanism. I propose and evaluate an alternative hypothesis: that circuit exposure itself, independent of any solute or cell removed, is immunomodulatory, and that this effect contributes additively to the clinical response seen in some apheresis indications. This idea has a documented history in bioengineering, dating to observations of complement-mediated leukopenia in early hemodialysis and the 1980s concept of “procedurally induced immunomodulation.” I synthesize supporting evidence across three lines: direct biocompatibility data showing that blood-membrane contact alone induces immunomodulation; a candidate molecular mechanism, soluble HLA class I adsorption to circuit surfaces, reported in a small study of modern apheresis procedures in patients and healthy donors; and incidental findings from the sham arms of two rheumatoid arthritis trials, in which circuit exposure without net removal was accompanied by immunological change, with inconsistent clinical response. I situate this evidence against the better-characterized precedent of circuit-induced immune activation in ECMO and cardiopulmonary bypass, while accounting for the substantial dose difference and anticoagulant differences between these modalities and apheresis. I propose a threshold model in which circuit-driven immunomodulation should vary by disease and immune activation state, and I outline a direct empirical test: prospective collection of immune biomarkers in both arms of future sham-controlled apheresis trials. This hypothesis is falsifiable within existing trial infrastructure and could inform how apheresis’ therapeutic mechanism, and its appropriate sham controls, are understood.

## Introduction

Therapeutic apheresis is conventionally understood through the lens of what it removes: pathogenic autoantibodies, immune complexes, cytokines, or lipoproteins. Recent reviews of therapeutic plasma exchange continue to attribute its immunomodulatory effects to removal of autoantibodies, immune complexes, complement and cytokines, and to replacement-fluid effects (1). This framework treats the extracorporeal circuit itself as an inert conduit: a delivery mechanism with no independent biological effect. That assumption is worth revisiting, and it has a longer history of being questioned than is generally appreciated.

The idea that an extracorporeal circuit might have biological effects of its own, independent of whatever it is designed to remove, originates in hemodialysis. Early clinical observations that dialysis produced transient leukopenia apart from any solute clearance were reported by the late 1960s, and by 1977 this was tied mechanistically to complement activation by the dialyzer membrane itself, the first clear demonstration that a synthetic blood-contacting surface could trigger a systemic immune reaction on its own (2). Bioengineers working through the 1980s built directly on this finding. Nosé and colleagues introduced the term ‘procedurally induced immunomodulation’ in 1989 to describe immunological effects produced by a device or method that were distinct from its intended therapeutic action, formalizing this into a broader concept of extracorporeal biomodulation and arguing that circuit material could be chosen deliberately for its biomodulatory properties rather than for biocompatibility alone (3, 4). By 1997, this reasoning had reached a striking conclusion: rather than treating procedurally induced immunomodulation purely as a side effect to be engineered away, Nosé's group proposed that apheresis systems could be deliberately designed to augment circuit-driven immunoactivation or immunosuppression as an additive therapeutic strategy in its own right (5).

This line of thinking never entered mainstream apheresis practice. But its central mechanistic claim, that circuit contact is immunologically active independent of removal, has direct modern support. Murabayashi and colleagues catalog material-dependent complement activation and leukocyte changes during membrane plasmapheresis itself (4), and more recently Ghio and colleagues identified a specific molecular pathway: soluble HLA class I molecules adsorb to apheresis circuit polymer surfaces during routine procedures, producing measurable immunomodulation in both patients and donors (6, 7). Taken together, this body of work suggests that the apheresis circuit is not simply an inert conduit for solute or cell removal, but an active immunological participant in its own right.

This manuscript examines whether circuit-induced immunomodulation could function as an additive therapeutic mechanism in apheresis; a contribution separate from, and alongside, whatever is removed. I consider the biological plausibility of this mechanism, why its magnitude should be expected to vary by disease and immune activation state, and how it is grounded in evidence from both modern and historical extracorporeal circuits. This manuscript addresses removal-based apheresis modalities. Extracorporeal photopheresis is excluded because its immunomodulatory action derives from a deliberate photochemical modification of the treated cells, which is a different mechanism from circuit exposure.

## Possible mechanisms for circuit-induced immunomodulation

The clearest evidence that blood-material contact alone can produce lasting immune changes comes from a systematic characterization of membrane plasmapheresis circuits (4). Complement activation during passage through the plasma separator generates C3a and C5a at levels that vary substantially by membrane material (8), and this activation is accompanied by transient leukopenia from pulmonary leukosequestration, a phenomenon first described in hemodialysis and traceable to the same complement-mediated mechanism (2). Circuit contact also triggers interleukin-1 release from mononuclear phagocytes, demonstrated even in an animal model with no underlying disease process to confound the result, again with the magnitude differing by membrane type (4). Perhaps most tellingly, when patient plasma collected before treatment was used to culture donor lymphocytes, it suppressed their response to mitogen stimulation below what was seen with normal control plasma; after plasmapheresis, the same patient's plasma no longer produced this suppression, even relative to control (4). Because this change reflects an alteration in what the plasma itself was doing to lymphocyte function, not simply a reduction in solute concentration, the authors concluded that the procedure was modulating immune reactivity through mechanisms beyond removal alone. Underlying much of this activity is a simple physical process: plasma proteins adsorb onto the membrane surface, can undergo conformational change or denaturation, and may later desorb back into circulation in an altered form capable of being recognized by phagocytic cells as foreign material (9).

The preceding evidence refers largely to membrane apheresis, although the mechanism could still apply to centrifugation-based systems. But recent studies suggest a mechanism that would directly apply to centrifugation apheresis. Ghio and colleagues found that soluble HLA class I (sHLA-I) molecules adsorb to apheresis circuit polymer surfaces during every procedure in patients with either CIDP or autoimmune disease associated with cryoglobulinemia, and that T lymphocytes and neutrophils passing through the circuit could plausibly bind these adsorbed molecules, inducing TGF-beta1 upregulation and soluble Fas ligand release (6). In ten patients with CIDP or cryoglobulinemia-associated vasculitis, markers of T-cell and neutrophil activation and TGF-*β*1 upregulation in CD8+ T cells and neutrophils persisted and accumulated across three procedures (6), whereas in 20 healthy donors the same pathway produced short-lived changes that returned to baseline by 14 days (7). This is a comparison between healthy donors and two disease states across two separate studies with different protocols, not a demonstrated gradient across the full range of conditions treated with apheresis, and it should be read as establishing a mechanism capable of producing activation-dependent variation rather than as proof that this specific pathway explains the full pattern of sham response seen in Table 1. It is, at minimum, a plausible basis for expecting that circuit-driven immunomodulation would be more pronounced in chronically activated, actively autoimmune patient populations than in healthy donors, and a starting point for testing whether the same gradient holds across a broader range of disease states than have so far been examined directly.

**Table 1:** Sham-controlled apheresis trials: modality, procedural descriptions, and sham arm outcomes.

Trial (year)	Modality	Condition	Sham procedure	Sham arm outcome	Notable sham arm finding
Dwosh 1983 (21)	TPE	RA	Circuit; plasma separated, recombined	Transient improvement similar to active arm	Both arms continued usual therapy; authors note possible plasticware adsorption of mediators causing response in sham arm
Canadian Cooperative MS Study Group 1991	TPE	CPMS	Circuit	No difference in treatment failure vs. active arms	Sham arm received placebo medications
Miller 1992 (23)	TPE + LCA	PM/DM	Cell separator; fractions recombined	3/13 improved, same as both active arms	PE and LCA changed labs (*P* < 0.001, *P* = 0.002) but neither beat sham clinically
Hahn 1996 (24)	TPE	CIDP	Circuit; blood recombined, reinfused	No improvement	Near-zero sham response; clear PE superiority
Weinshenker 1999 (25)	TPE	Acute CNS demyelinating	Circuit; blood recombined, reinfused	1/17 (6%) improved	Low but non-zero sham response
Brochet 2020 (26)	TPE	MS relapses	Full circuit; own plasma reinfused	17.6% full recovery at 1 month	Best-documented sham procedure; TPE favored on secondary outcomes
España-Cueto 2025 (27)	TPE	Post-COVID	Spectra Optia	No improvement	Neither sham nor active arm improved
Wallace 1982 (28)	LCP	RA	Full circuit; no net cell removal	Ritchie Index improved (*P* < 0.01)	Objective improvement in sham arm; usual medications continued
Verdickt 1983 (14)	LCP	RA	Full circuit; no net cell removal	Significant improvements in Ritchie index, pain relief, and grip strength	Usual medications continued
Felson 1999 (29)	Protein A IA	RA	Full circuit; column bypassed	11.4% ACR response	Equal arthralgias in both arms
Hidaka 1999 (15)	Filtration LCA	RA	Sham column; matched circuit exposure	0/7 achieved ACR 20%	Usual medications continued in both arms
Gendreau 2001 (30)	Protein A IA	RA	Full circuit; column bypassed	15.6% ACR response	Analysis of patients who completed all treatments
Sawada 2005 (31)	LCAP	UC	Sham device; full circuit	33% improved by CAI	More adverse events than active arm; usual medications continued in both arms
Sands 2013 (32)	GMA	Crohn's disease	Full circuit; column bypassed	19.2% remission	Matched active arm (17.8%); identical adverse events
Khan 2017 (33)	LA	Refractory angina, elevated Lp(a)	Needle only; not connected to machine	No change in MPR or endpoints	Only true venipuncture sham; no circuit exposure

TPE, therapeutic plasma exchange; LCP, lymphoplasmapheresis; LCA, leukapheresis; LCAP, leukocytapheresis (Cellsorba column); GMA, granulocyte/monocyte apheresis (Adacolumn); Protein A IA, protein A immunoadsorption (Prosorba column); LA, lipoprotein apheresis; RA, rheumatoid arthritis; CPMS, chronic progressive multiple sclerosis; MS, multiple sclerosis; PM/DM, polymyositis/dermatomyositis; CIDP, chronic inflammatory demyelinating polyneuropathy; UC, ulcerative colitis; CAI, clinical activity index; ACR, American College of Rheumatology; MPR, myocardial perfusion reserve; PE, plasma exchange.

Mechanical shear stress is itself an independent modulator of leukocyte behavior, separate from any chemical or material property of the circuit. Under normal physiological flow, shear stress helps keep circulating neutrophils and monocytes in a quiescent state; this deactivating effect is disrupted in disease states such as hypertension and hypercholesterolemia, leaving a larger population of circulating leukocytes in an activated, adherent phenotype (10). Supraphysiological shear, of the kind generated by mechanical circulatory support devices, pushes leukocyte behavior in the same direction from the opposite end: it damages neutrophil membranes, sheds surface receptors including CD62L, and drives aberrant activation markers such as CD11b upregulation (11). Critically, shear stress does not act in isolation in an extracorporeal circuit; it acts concurrently with blood-material contact, and the combination of the two produces leukocyte changes beyond what either exposure produces alone (12). This shear-material interaction has been documented in the same class of devices already discussed above: cardiopulmonary bypass and ECMO circuits combine substantial shear forces with continuous biomaterial contact, and immune dysfunction in patients on these devices reflects the joint contribution of both factors rather than either mechanism in isolation (13).

Direct clinical corroboration of the above phenomena can be found within the sham arms of two rheumatoid arthritis (RA) apheresis studies themselves. In a double-blind trial of lymphoplasmapheresis, the control procedure consisted of whole blood diverted to a centrifuge bowl, separated into plasma and cellular fractions, and immediately reinfused in total — a design with no net solute or cell removal (14). This sham arm nonetheless showed a significant fall in serum IgA, and a significant rise in circulating immune complexes, with statistically significant clinical improvement in several parameters. Similarly, a sham-controlled trial of filtration leukocytapheresis for RA, the control procedure used a hollow column identical in volume to the active adsorption column, perfused at matched flow and duration but without a leukocyte-trapping surface (15). In a later sub-study of this trial, Hidaka et al. found that despite the absence of any cell removal, the sham arm showed significant increases in synovial fluid IL-10, significant decreases in synovial fluid MCP-1, and a decline in synovial fluid RANTES of a magnitude comparable to the active arm (16). However, these cytokine changes occurred without clinical response: none of the seven sham-arm patients achieved an ACR20 response in the parent trial (15). Although the N was small in both studies, these findings, arising incidentally within trials designed to test a removal-based hypothesis, are consistent with the broader biocompatibility literature and support the conclusion that circuit exposure itself is not immunologically inert.

Adsorptive column devices show that blood–material contact can drive specific immunological responses beyond cell removal. In granulocyte/monocyte adsorption, myeloid cells bind to the carrier surface and undergo release reactions, including shedding of L-selectin and TNF receptors, with soluble forms detected in the column outflow returned to the patient (17). *In vitro*, exposing heparinized healthy-donor blood to the carrier produced L-selectin shedding, reactive oxygen species production, neutrophil apoptosis, and degranulation, and in rabbits extracorporeal circulation through the column desensitized neutrophil chemotaxis to IL-8 (18). The proposed route is ligation of neutrophil Fc and complement receptors by immunoglobulin and complement fragments deposited on the carrier, producing a response resembling phagocytosis-induced cell death (18). The effects of these devices therefore cannot be attributed to removal alone. This is contact with a purpose-built adsorbent and not with unmodified tubing or membrane, so I do not offer it as evidence for an effect of an ordinary apheresis circuit. It shows that the mechanism class, material-driven activation of complement and leukocytes with downstream regulatory consequences, can have measurable immunological consequences.

Studies of regular apheresis donors do not show significant immunological changes, but this does not argue against a circuit effect in patients. In regular plateletpheresis donors followed for up to 12 months, lymphocyte subsets, serum proteins, and ferritin did not change significantly (19), and a donor-safety review found no evidence of increased infection and described immune-responsiveness data as generally reassuring (20). These findings concern healthy people, whose immune cells and complement system are not primed by disease, and they do not show whether the same exposure has an effect in patients with active inflammation. As one illustration of how baseline immune state differs, pro-inflammatory CD14 + CD16 + monocytes make up about 8%–10% of monocytes in healthy persons and are expanded in patients with active inflammatory bowel disease and generalized pustular psoriasis (17). In the donor studies, the endpoints were also mostly lymphocyte subsets measured at intervals of months, not complement or neutrophil activation near the time of the procedure. Donors are also a selected group of repeat attenders, and attrition in Hans 2023 left 10 of 33 donors at the final time point. The donor data therefore set no upper bound on the effect of circuit exposure in patients. They indicate that any such effect is small or transient in healthy people, and they leave the question open for disease states.

## What the sham-controlled trial literature illustrates

If circuit exposure is immunologically active in apheresis procedures, the clearest place to look for its footprint is the sham arm of a controlled apheresis trial: the one context in which patients undergo the extracorporeal procedure without the intended therapeutic removal. I use this literature here as illustrative support for the biological plausibility of an additive circuit mechanism, not as a basis for judging whether therapeutic plasma exchange or any other apheresis modality worked in any of the trials discussed below. I do not argue that these trials should be reinterpreted, that their conclusions were wrong, or that TPE is or is not effective in the diseases studied. The trials were not designed to detect a circuit effect, and none of them can settle the question of whether one exists. What they can do is show what a sham apheresis procedure actually involves biologically, which is the relevant question here.

Table 1 summarizes sham procedures and outcomes across fifteen sham-controlled apheresis trials spanning four decades, several disease categories, and multiple apheresis modalities (14, 15, 21–33). Trials were identified by sequential searches of multiple literature databases. Trials in which the sham arm received a new, active immunosuppressive therapy were excluded. Trials in which the sham arm continued usual medications to which the patients were not responding were included. In every trial but one, the sham arm involved genuine extracorporeal circuit exposure: blood was drawn, processed through the apheresis device, and returned, differing from active treatment only in replacement fluid or the component discarded.

Two patterns in this sample are worth attention, with one caveat on composition: six of the fifteen trials are in rheumatoid arthritis, reflecting how often RA has served as the testing ground for sham-controlled apheresis historically rather than any deliberate weighting in trial selection. The following observations about cross-disease variation should be read with that concentration in mind. First, sham-arm response varies considerably across conditions, from near-zero in chronic inflammatory demyelinating polyneuropathy (CIDP) to more substantial signals in other conditions. If circuit-induced immunomodulation is real, it should not be expected to contribute equally across conditions. A threshold model is more plausible than a uniform one: non-specific immune modulation from circuit contact would be expected to matter most in conditions where the underlying immunopathology is diffuse or partially amenable to non-specific dampening, and least in conditions driven by a single, high-titer, well-characterized pathological factor. This threshold model is offered as an organizing principle rather than a tested hypothesis; the trials summarized here were not designed to test it.

Sham response rates in Table 1 cannot by themselves indicate a circuit effect. A sham arm is a placebo arm, and improvement may reflect placebo response, natural history, or regression to the mean in patients enrolled during a flare. Where sham responses can be compared with pooled placebo rates from drug trials in the same disease, they are at or below those rates (34, 35). These comparisons are imperfect because the trials differ in populations, eras, endpoint definitions, and blinding, and because a procedural sham may elicit a larger placebo effect than an inert pill (36). Sham responses that match or fall below placebo rates do not exclude a circuit effect, since a circuit-driven immunological change may occur without producing a clinical response (as in the Hidaka sham arm) and these trials were not designed to detect it. The hypothesis proposed here is therefore not a claim about clinical response rates. It predicts measurable biological changes during circuit exposure without net removal, and it is tested by collecting such biomarkers in both arms of future trials.

The Miller 1992 three-arm trial in polymyositis and dermatomyositis illustrates the interpretive problem. Plasma exchange reduced serum muscle enzymes and leukapheresis reduced lymphocyte counts, yet 3 of 13 patients improved in each of the three arms, including the circuit-exposed sham arm (23). This pattern is compatible with several explanations: no clinical effect of either intervention, placebo response, natural history, or a contribution from circuit exposure. The trial cannot distinguish among them (23).

Khan 2017 provides the one contrast in Table 1 (33). In this single-blinded crossover trial of lipoprotein apheresis for refractory angina (*n* = 20), patients in the control arm attended weekly sessions for three months in the apheresis unit, had a needle inserted, and were blinded with screens and drapes while the machine was run to simulate treatment, but the tubing was not connected and no blood was processed. Attendance at sham sessions was 96%. Despite this, sham-arm myocardial perfusion reserve did not improve (−0.16; 95% CI −0.33 to 0.02), and neither did exercise capacity nor symptom scores, whereas the active arm improved on most of them (33). The elements shared by every sham in the table, including repeated attendance, clinical attention, needle insertion, expectation, and blinding, were therefore not sufficient to produce a response in this trial. This is consistent with circuit blood contact, and not these non-specific elements alone, contributing to the responses seen in circuit-exposed shams elsewhere, although it cannot conclusively demonstrate that. It should also be stated that the sham arm differed from active treatment in more than circuit contact. I therefore offer it as a constraint on non-specific explanations, and not definitive proof of the circuit contact mechanism.

Finally, one of the earliest trials in this literature noticed something themselves, well before any mechanistic pathway had been described. In Dwosh 1983, patients in the sham plasmapheresis arm for rheumatoid arthritis improved to a similar degree as the active arm, and although the authors thought a placebo response was the most likely mechanism, they did also propose that adsorption of inflammatory mediators onto the circuit's plasticware surfaces was a plausible explanation (21). This observation was not a reinterpretation imposed after the fact: it was made by the original investigators, in real time, decades before Ghio's sHLA-I pathway gave the phenomenon a name. That this independent trial team suggested a circuit-based explanation of their own sham-arm findings is an early suggestion consistent with this mechanism that predates modern literature entirely.

## Comparison to ECMO and CPB, and the question of modern circuits

The biological plausibility of circuit-induced immunomodulation does not rest on the apheresis literature alone. It is most extensively characterized in ECMO and cardiopulmonary bypass (CPB), where circuit-induced immune activation is a well-recognized clinical problem occurring independent of any therapeutic removal. CD18 upregulation and L-selectin shedding on neutrophils and monocytes occur within two to four hours of ECMO circuit exposure (37). Neutrophil elastase and lactoferrin are released during cardiopulmonary bypass at levels significantly exceeding unexposed controls (38). These effects are attributable specifically to blood-material contact.

Apheresis operates at substantially lower intensity than either. ECMO runs continuous flows of 3–5 L/min for days to weeks; CPB runs 2–5 L/min for hours, often with hypothermia and cardiotomy suction; therapeutic apheresis typically runs 50–150 mL/min for two to four hours (Table 2). This is a real difference in dose, not a rhetorical one, and any claim that apheresis circuits produce meaningful immunomodulation must account for it rather than gesture at a vague continuum. However, Ghio et al. provides recent examples of apheresis circuits producing measurable adherence of sHLA (6, 7); and older literature supports the concept that apheresis circuitry can induce changes in immunological mediators as discussed above (14, 16).

**Table 2:** Circuit dose intensity across extracorporeal modalities.

Modality	Typical blood flow	Typical duration	Notes
ECMO	3–5 L/min	Days to weeks	Continuous circuit exposure; largest cumulative blood-material contact
Cardiopulmonary bypass	2–5 L/min	Hours	Often combined with hypothermia and cardiotomy suction, both independently activating
Dialysis	300-500 mL/min	3-5 h per session	Intermittent sessions; presence of dialysis membrane
Therapeutic apheresis	50–150 mL/min	2–4 h per session	Intermittent sessions; lowest flow and shortest single-session duration

Another reasonable objection is that this evidence is dated: much of the classic complement-activation and leukocyte-activation literature comes from 1980s-to-2000s membranes, at a time when circuit biocompatibility was considerably worse than it is today. Modern apheresis tubing has moved toward non-DEHP formulations and more refined polymer surfaces, and this improvement is real. But two points limit how much weight this objection can bear. First, a 2023 review of complement activation in extracorporeal circuits explicitly lists plasmapheresis, alongside hemodialysis, hemofiltration, ECMO, and CPB, as a modern treatment modality in which the alternative pathway amplification loop remains essentially unregulated on any synthetic surface. Material refinement changes the magnitude of activation, not its presence (39). Second, and more directly relevant, the sHLA-I adsorption mechanism was not a historical artifact: it was demonstrated using contemporary apheresis platforms and circuits in current clinical use, in both donor and patient populations, within the past fifteen years (6, 7). The claim that circuit biomodulation is a phenomenon of obsolete materials is not supported by the most mechanistically specific data available.

A further difference between apheresis and the ECMO and CPB literature is anticoagulation. ECMO and CPB circuits are conventionally heparinized, and the membrane plasmapheresis study that anchors much of the mechanistic case was also performed with heparin (3, 4). Centrifugal apheresis, by contrast, typically relies on citrate, which chelates ionized calcium in the circuit and thereby reduces calcium-dependent blood-surface interactions. Relative to heparin, citrate has been associated with less complement activation and neutrophil degranulation. In a single-patient comparison of membrane plasma separators, citrate produced less cellular and complement change than heparin (40), and in critically ill patients on hemofiltration, heparin but not citrate was associated with net generation of C5a, elastase, and myeloperoxidase across the filter (41). These data are limited in size and design, and most come from hemofiltration or dialysis rather than apheresis. They indicate attenuation rather than elimination of circuit-induced activation. Complement and platelet activation remained detectable under citrate in an *in vitro* dialysis model, although that model also contained low-dose heparin (42), and complement activation persisted on a cellulose diacetate membrane with citrate in the single-patient plasmapheresis comparison (40). The distinction also does not follow modality strictly, since membrane plasmapheresis has typically been heparinized and citrate is now widely used in continuous renal replacement therapy. I therefore treat anticoagulant as a modifier of circuit-driven immunomodulation rather than a reason to dismiss it. This widens the dose gap described above, and it means the ECMO and CPB comparison supports the plausibility of the mechanism class, not its magnitude in citrate-anticoagulated apheresis. Whether the sHLA-I adsorption pathway (6, 7) depends on anticoagulant has, to my knowledge, not been tested.

## Discussion

None of this establishes that circuit exposure is therapeutic in any specific apheresis indication. What it supports is biological plausibility: a mechanism class with a clear analog in ECMO and CPB (at higher intensity and, in most cases, under heparin); a candidate pathway, sHLA-I adsorption, reported in a small study of modern apheresis procedures, with changes accumulating across repeated procedures in patients and transient in healthy donors; and a pattern in the sham-controlled literature that is consistent with, though not evidence for, an additive circuit effect that may vary by disease.

This hypothesis is testable within the framework of current knowledge, not merely a plausible narrative. If circuit-driven immunomodulation is real and additive, prospective trials that collect immune biomarkers, complement activation products, leukocyte activation markers, sHLA-I levels, in both active and sham arms should find measurable change in the sham arm that correlates with clinical outcome, and the magnitude of that change should track disease and baseline immune activation state along the lines proposed here. Because anticoagulant modifies circuit-induced activation, such trials should record the anticoagulant and platform, and circuit-driven biomarker changes would be predicted to be larger under heparin than under citrate. A finding of no measurable immunological change in genuinely circuit-exposed sham arms, replicated across conditions, would argue against the hypothesis as framed. This is a stronger test than the trial literature summarized in Table 1 can offer on its own, since those trials were not designed to isolate a circuit effect from other sources of sham-arm response such as regression to the mean or non-specific placebo effect.

Several limitations bear on how much weight this hypothesis should carry at this stage. The sham-controlled trial literature summarized here is illustrative, not an exhaustive or systematically ascertained sample, and it is weighted toward rheumatoid arthritis for historical reasons rather than any biological reason to expect the effect to concentrate there. The sHLA-I pathway, the most mechanistically specific evidence available, has so far been demonstrated in two disease states and in healthy donors; whether it generalizes across the fuller range of conditions treated with apheresis is untested. Table 1 does not record the apheresis platform (membrane or centrifugal) or the anticoagulant (citrate or heparin), because the source trials do not reliably report them. I therefore could not examine whether sham-arm immunological or clinical responses varied with either. Both are plausible modifiers of circuit-driven activation, so the trials may differ in circuit exposure in ways this manuscript cannot account for, and future sham-controlled trials should report both. And the dose gap between apheresis and the ECMO/CPB literature that anchors much of the mechanistic case is real: any claim that circuit contact drives clinically meaningful immunomodulation at apheresis-level flow and duration remains an extrapolation until tested directly in apheresis circuits themselves.

If this mechanism is real, therapeutic apheresis’ clinical effect in some conditions may be a composite of two contributions rather than one: solute or cell removal, and circuit-driven immunomodulation. This has practical implications beyond mechanism. It suggests that sham arms in apheresis trials should not be treated as biologically neutral controls by default, a design assumption embedded in how such trials are typically interpreted; and it suggests that the contribution of circuit exposure to sham-arm responses should be considered, alongside placebo response and regression to the mean, when interpreting ambiguous trials. Neither point requires revisiting the conclusions of any completed trial. It does suggest a concrete next step: prospective collection of the biomarkers above in both active and sham arms of future apheresis trials, across a range of conditions with differing immunopathology. That would allow the additive-mechanism hypothesis to be tested directly, rather than inferred from the pattern of results left behind by trials that were never designed to look for it.

## Data Availability

The original contributions presented in the study are included in the article/supplementary material, further inquiries can be directed to the corresponding author.
